# Dr. Reynell Coates to the Editors

**Published:** 1842-04-23

**Authors:** Reynell Coates

**Affiliations:** Philadelphia


To the Editors of the Medical Examiner.

Gentlemen,—Though induced by considerations of a private nature to resign the immediate responsibility of co-editorship with you, permit me to express my readiness to serve the interests of your Journal, to the extent of my ability, in any manner which you may deem desirable.

I regret that this offer is, at best, but a poor return for the uniform courtesy and kindness received at your hands during our short term of official association. In the hope that this change of relation may render my humble labours rather more than less useful to yourselves and your subscribers, I forward you another of the series of surgical notes commenced in a recent number of the Examiner.

Yours, very sincerely,
REYNELL COATES.

*Philadelphia, April 12th,* 1842.

